# What is the perception of biological risk by undergraduate nursing students?

**DOI:** 10.1590/1518-8345.0722.2715

**Published:** 2016-07-04

**Authors:** Mª Carmen Moreno-Arroyo, Montserrat Puig-Llobet, Anna Falco-Pegueroles, Maria Teresa Lluch-Canut, Irma Casas García, Juan Roldán-Merino

**Affiliations:** 1 MSc, Professor, Escuela Universitaria de Enfermería, Universidad de Barcelona, Barcelona, Spain.; 2 PhD, Professor, Escuela Universitaria de Enfermería, Universidad de Barcelona, Barcelona, Spain.; 3 PhD, Full Professor, Escuela Universitaria de Enfermería, Universidad de Barcelona, Barcelona, Spain.; 4 PhD, Professor, Hospital Germans Trias i Pujol de Badalona, Barcelona, Spain, and Universitat Autônoma de Barcelona, Barcelona, Spain.; 5 PhD, Full Professor, Campus Docent Sant Joan de Déu, Fundació Privada, Escuela Universitaria de Enfermería, Universidad de Barcelona, Barcelona, Spain.

**Keywords:** Perception, Risk Factors, Students, Nursing

## Abstract

**Objective:**

to analyze undergraduate nursing students' perception of biological risk and its relationship with their prior practical training.

**Method:**

a descriptive cross-sectional study was conducted among undergraduate nursing students enrolled in clinical practice courses in the academic year 2013-2014 at the School of Nursing at the University of Barcelona. Variables: sociodemographic variables, employment, training, clinical experience and other variables related to the assessment of perceived biological risk were collected. Both a newly developed tool and the Dimensional Assessment of Risk Perception at the worker level scale (*Escala de Evaluación Dimensional del Riesgo Percibido por el Trabajador*, EDRP-T) were used. Statistical analysis: descriptive and univariate analysis were used to identify differences between the perception of biological risk of the EDRP-T scale items and sociodemographic variables.

**Results:**

students without prior practical training had weaker perceptions of biological risk compared to students with prior practical training (p=0.05 and p=0.04, respectively). Weaker perceptions of biological risk were found among students with prior work experience.

**Conclusion:**

practical training and work experience influence the perception of biological risk among nursing students.

## Introduction

One of the main objectives of university education is to prepare students for the professional world and to enable them to develop the skills that define each discipline. In the field of health sciences, training includes interventions aimed at the acquisition of knowledge, skills and attitudes needed to be a competent health professional. In this environment, there must be skills aimed at promoting and ensuring the safety of the student and the patient. 

Healthcare professionals are exposed to numerous risks^1^, and biological risk is one of the most important risks due to its severity and increasing frequency^2^ ^-^ ^3^. Biological risk is an important issue in public health, and although hepatitis B, hepatitis C and Human Immunodeficiency Virus (HIV) infections are the most well known, other emerging diseases (e.g., Ebola) can be acquired by other routes of biological exposure, such as air or physical contact.

In this context, undergraduate nursing students work in an unfamiliar and complex clinical environment that exposes them to numerous risks^4^ ^)^ during their formative years. Their inexperience^1^ ^)^ and stress levels^5^ ^-^ ^10^ are compounded with changing situations and constant uncertainty in this environment.

Several studies on accidents involving nursing students during the course of their clinical practice have shown that rates of biological risk exposure (e.g., punctures, eye splashes and cuts) remain very high^11^ ^-^ ^15^. The EPINETAC project^3^ found that a considerable portion of percutaneous accidents is caused by inadequate maneuvers that are banned by standard recommendations, such as the recapping of needles. This finding shows significant deficiencies in security measures and points to low effectiveness of theoretical and practical training to prevent biological risks among college students. 

Additionally, nursing professionals perceive low student knowledge of protocols and preventive measures and student attitudes of poor initiative and insecurity^13^ ^-^ ^14^ ^,^ ^16^. These issues should be considered when planning educational activities for students.

A multicenter study^17^ ^)^ on the use of standard precautions against biological agents showed a high degree of conceptual confusion and a lack of awareness of preventive measures, and this study found risk behaviors related to protections used by participants from different healthcare fields. Another study^18^ determined that training for standard hygienic and precautionary measures was not universally performed over all studies, and some discrepancies existed between theoretical and practical training.

According to the legal regulations in place in Spain for the prevention of risks (Law 31/1995 of 8 November on Prevention of Occupational Risks), health institutions are committed to promoting a culture of prevention among workers, but few provisions include trainees. Only RD 783/2001 includes the protection of trainees in ionizing radiation.

Another important issue related to prevention is an individual's perception of risk. Some authors state that this perception affects one's attitude towards risk and one's behavior at work^19^ ^-^ ^21^. In the study by Cordeiro^22^ ^)^ that examined this relationship between risk perception and the likelihood of suffering an occupational accident, accident victims were those with the lowest perception of risk. 

Despite the importance of biological risk to healthcare staff, few studies have examined factors related to risk perception among either health professionals or undergraduate nursing students. Thus, this study aims to analyze undergraduate nursing students' perception of biological risk and its relation to previous practical training.

## Method

This descriptive cross-sectional study was conducted at the University School of Nursing (EUE) at the University of Barcelona (UB) from September to December 2013.

The study population consisted of undergraduate nursing students at the UB enrolled in clinical practice subjects during the academic year 2013-2014. Two groups of students formed the sample. Group 1 had no prior clinical practice training (second-year students have not undergone any practical training). Group 2 had previous clinical practice training (third-year students have undergone a previous period of external academic clinical practice).

The inclusion criteria in both groups included having passed all the basic training and compulsory subjects of the first-year nursing degree and being enrolled in the second-year practical subjects (Group 1) or in the third-year nursing degree (Group 2). All students who refused to participate in the study were excluded.

Ultimately, the sample consisted of 78 students (37 without prior practical training and 41 with prior practical training).

### Study variables

*-Variables related to sociodemographics, employment, training on biological risk prevention and students' immunization status*: These variables included age; sex; prior practical training; admission procedure to the undergraduate nursing degree; previous work experience in the health field; current work experience; having completed external courses on the prevention of biological risk; vaccination against hepatitis B, hepatitis A and tetanus; and having received the tuberculin test. 

*-Variables related to the dimensional assessment of perceived biological risk:* These variables included students' perceptions of the following: emotional response to fear; vulnerability; severity of the consequences; control/doom (i.e., whether the student believes in his capacity to affect preventive actions); degree of control; catastrophic potential attributed to the risk factor; delayed consequences; and perceived risk magnitude. All of these variables were measured using the Dimensional Assessment of Risk Perception at the worker level (Escala de Evaluación Dimensional del Riesgo Percibido por el Trabajador, EDRP-T)^23^.

### Tool

A 2-part data collection sheet was designed as follows:

- Section A: This form that included variables related to the following characteristics: sociodemographics, work, training on the prevention of biological risks prior to starting the undergraduate degree, training on clinical practices performed during undergraduate studies and students' vaccination status.

- Section B: This form included the EDRP-T scale, which consisted of 10 questions that aimed to evaluate students' perceptions of biological risk. This evaluation scale is part of the Technical Note on Prevention (NTP) 578 published by the Spanish National Institute of Safety and Health at Work (INSHT) in 2001^23^. The scale is a flexible evaluation tool that can be adapted to different types of risks, and thus the guidelines used by the authors were adapted to evaluate students' perceptions of biological risk. The first 9 questions are evaluated on an ordinal scale of 1 to 7 points, where 1 is the lowest, and 7 is the highest. Question 10, which evaluates the overall magnitude of the biological risk, is assessed with a discrete quantitative scale of 0-100, where 0 represents very low risk, and 100 represents very high risk.

The first 2 questions of this section (B1 and B2) explore the knowledge among students and among the professional nurses responsible for these students. Question B3 explores the emotional response to fear, which was considered by the authors to be the most predictive of overall perceived risk. Question B4 assesses the one's feelings of vulnerability or susceptibility. Question B5 explores the perception of the severity of the consequences. Questions B6 and B7 are related to the perception of control/fatality of the risk and explores students' ability to perform both preventive and protective actions. The authors believe that the perception of control, as assessed by question B7, can cause feelings of invulnerability, as assessed in question B4. Question B8 explores the catastrophic potential attributed to the biological risk factor, which is an issue related to the perceived overall risk (B10). Question B9 regards the perception of delayed consequences, and question B10 aims to obtain an overall estimate of the magnitude of the perceived biological risk. 

### Procedure

Data collection began after informing both the teachers responsible for the practical subjects and the students enrolled in these subjects of the study. The researchers provided the questionnaire, and participants in each group completed the questionnaire during an established period. The first questionnaire was completed by Group 1 (students without previous training in clinical practice) in October. The second questionnaire was completed by Group 2 (students with previous practical training) in November. In both cases, data collection was conducted during the briefing, which was one week prior to the start of clinical practice in the centers.

### Statistical Analysis

A descriptive analysis of all variables included in the study was performed. Frequencies and percentages of each qualitative variable were calculated, as were the mean and standard deviation of each quantitative variable. Differences between students with and without practical training were assessed by the chi-square test or Fisher's exact test for qualitative variables and by Student's t test or the Mann-Whitney U test for quantitative variables. 

An analysis was also performed to identify differences between the perception of biological risk of each item on the EDRP-T scale and sociodemographic variables using the Mann-Whitney U test or the Kruskal-Wallis test. A bilateral p = 0.05 was considered statistically significant. PASW Statistics v.20 software was used. 

### Ethical issues

Authorizations from both the Bioethics Committee of the UB and the direction of the EUE were obtained. Students were provided with verbal and written information about the study to ensure the anonymity and confidentiality of data. 

## Results

Of 40 students in Group 1 (those without prior practical training) and 49 students in Group 2 (those with prior practical training) who were available during administration of the questionnaire, 38 and 45 students, respectively, met the inclusion criteria. Ultimately, 37 students in Group 1 and 41 students in Group 2 participated, yielding a total sample of 78 students.

### Sociodemographic, work and educational with regard to occupational risk prevention and clinical (vaccination status) features of the students

A total of 61.5% of students were less than 25 years of age, and 80.7% were women. A total of 56.4% of students were admitted to the nursing degree through high school.

With regards to work experience in the health field, 69.2% of students had no previous experience, and only 11.5% were working at the time of the study. A total of 23.1% of students had undergone training on occupational risk prevention prior to beginning their degree. With regards to vaccination status, most students reported being properly vaccinated against hepatitis A (93.6%), hepatitis B (97.4%), and tetanus (94.9%), but only 76.9% of students reported undergoing the tuberculin test.

No statistically significant differences were found between Group 1 and Group 2 for any variables. Sociodemographic, employment, training (occupational risk prevention) and vaccination status characteristics are shown in Table 1.

**Table 1:** Sociodemographic, employment, training (on prevention of occupational risk prevention) and vaccination status of undergraduate nursing students; Barcelona, Spain, 2013

Items		Total		Prior practical training			P	
				Group 1 (without training)		Group 2 (with training)		
		n	%	n	%	n	%	
Categorized age								
	Less than 25 years of age	61	78.2	31	83.7	30	73.2	.321^*^
	Between 25 and 35 years of age	14	17.9	4	10.8	10	24.4	
	Older than 35 years	3	3.9	2	5.5	1	2.4	
Sex								
	Man	15	19.3	10	27.0	5	12.2	.097†
	Woman	63	80.7	27	73.0	36	87.8	
Admission to the degree studies								
	Training courses	30	38.5	13	35.1	17	41.5	.645†
	High school	44	56.4	22	59.5	22	53.7	
	Entrance exam among those > 25 years old	3	3.8	1	2.7	2	4.9	
	Other degrees	1	1.3	1	2.7	0	0.0	
Prior work experience								
	Yes	24	30.8	9	24.3	15	36.6	.241†
	No	54	69.2	28	75.7	26	63.4	
Mean months worked (standard deviation)		35.3 (20.0)		13.6 (42.9)		13.3 (22.9)		.241‡
Current work experience								
	Yes	9	11.5	4	4.3	5	4.7	.999^*^
	No	69	88.5	33	32.7	36	36.3	
Prior training on risk prevention								
	Yes	18	23.1	7	18.9	11	26.8	.408†
	No	60	76.9	30	81.1	30	73.2	
Hepatitis A vaccination								
	Yes	73	93.6	36	97.3	37	90.2	.213^*^
	No	5	6.4	1	2.7	4	9.8	
Hepatitis B vaccination								
	Yes	76	97.4	37	100	39	95.1	.273^*^
	No	2	2.6	0	0.0	2	4.9	
Tetanus vaccination								
	Yes	74	94.9	35	94.6	39	95.1	.999^*^
	No	4	5.1	2	5.4	2	4.9	
Tuberculin test								
	Yes	60	76.9	29	78.4	31	75.6	.675^*^
	No	15	19.2	6	16.2	9	22.0	
	Do not remember	3	3.8	2	5.4	1	2.4	

* Fisher's exact test

† Pearson's chi-square test

‡ Student's *t*-test Fisher's exact test

### Assessment of biological risk perceived by undergraduate nursing students

The relationship between the perception of biological risk and students' prior practical training was analyzed. Statistically significant differences were found for perception of knowledge of biological risk (B1) and the possibility of harm due to a biological agent (B4); students without prior practical training had weaker perceptions of knowledge of risks and damage due to biological risk than students with prior practical training (p=0.05 and p=0.04, respectively). 

Statistically significant differences were also found for items B8 (harm to a large number of people) and B10 (overall magnitude of perceived risk), although in this case, students without practical training had a stronger perceptions of the catastrophic potential attributed to the biological agent and the overall perception of biological risk than students with prior practical training (both p=0.05). 

The relation between the perception of the biological risk and the presence or absence of prior practical training among undergraduate nursing students is shown in Table 2.

**Table 2:** Relationship between the perception of biological risk and the presence or absence of prior practical training among undergraduate nursing students; Barcelona, Spain, 2013

Dimensional evaluation of perceived risk	Prior practical training					p	
	Group 1 (without training)			Group 2 (with training)			
	P25	P50 (Median)	P75	P25	P50 (Median)	P75	
Knowledge of students of the biological risk	4	5	6	5	6	6	.05*
Knowledge of persons-in-charge of the biological risk	4	6	6	4.5	6	7	.40*
Fear of harm derived from the biological accident	5	6	7	5	6	7	.23*
Possibility of harm derived from the biological agent	3	4	5	3.5	5	6	.04*
Severity of harm that may result	4.2	5	6	5	6	7	.08*
Extent to which the risk can be avoided	5	6	7	6	6	7	.23*
Possibility of control in a risky situation	4.2	5	6	4	5	6.5	.65*
Extent to which a large number of people can be harmed	4	5.5	7	3	4	6	.05*
Immediacy of consequences	3	4	5	4	5	6	.31*
Overall magnitude of the perceived biological risk	70.7 (14.9)			61.6 (23.7)			.05†

* Mann-Whitney U test

† Student's t-test Fisher's exact test

With regards to the sociodemographic, employment and training characteristics of the students, statistically significant differences were found between Group 1 and Group 2 for item B10 (overall perception of risk) and item B7 (the extent to which a large number of people can be harmed). Work experience was associated with a weaker perception of biological risk, while no work experience was associated with a stronger perception of the ability to prevent or reduce damage due to biological risk. 

Statistically significant differences were also found with regards to sex for items B1 (students' perception of knowledge of biological risk) and B5 (severity of the potential harm caused by the biological risks), with stronger perceptions of knowledge of risk and weaker perceptions of the severity of the consequences among men. Other items related to the perception of biological risk and sociodemographic, work and training characteristics of the students are described in Table 3. 

**Table 3:** Relationship between the perception of biological risk and the sociodemographic, employment and training features of undergraduate nursing students; Barcelona, Spain, 2013

	n	Total (B10)	B1	B2	B3	B4	B5	B6	B7	B8	B9
		Mean (SD)	P50 (P25- P75)	P50 (P25- P75)	P50 (P25- P75)	P50 (P25- P75)	P50 (P25- P75)	P50 (P25- P75)	P50 (P25- P75)	P50 (P25- P75)	P50 (P25- P75)
Categorized age*											
Less than 25 years of age	61	68.3 (17.9)	5 (4-6)	6 (4.5-6)	6 (5-7)	5 (3-5)	6 (5-6.5)	6 (5-7)	5 (5-6)	5 3-6)	4 (4-5.5)
Between 25 and 35 years of age	14	59.5 (25.2)	6 (4.5-6)	6 (4-7)	5.5 (5-7)	5 (3-6)	6 (5-7)	6 (6-7)	6 (3.5-7)	5 (2.5-6.25)	5 (3.5-6.2)
Older than 35 years	3	46.6 (35.1)	6 (3-6)	6 (4-6)	6 (2-6)	4 (2-4)	5 (4-5)	7 (3-7)	6 (5-6)	4 (1-4)	4 (3-4)
Sex^†^											
Man	15	66.7 (20.4)	6 (5-7)^‡^	6 (4-7)	6 (6-7)	5 (3-6)	5 (4-5) ‡	6 (5-7)	5 (4-6)	5 (4-7)	5 (4-6)
Woman	63	62.3 (20.6)	5 (4-6)	6 (4-7)	6 (5-7)	4 (3-6)	6 (5-7)	6 (5-7)	5 (4-6)	5 (3-6)	4 (4-5)
Prior work experience^†^											
Yes	24	60.2 (24.9)^‡^	6 (5-7)	6 (4-7)	6.5 (5.2-7)	5 (3.2-6)	5.5 (5-7)	6 (5-7)	6 (5-7)^‡^	4 (2.2-7)	4 (3.2-6)
No	54	68.5 (17.7)	6 (4.7-7)	6 (5-7)	6 (5-7)	4.5 (3-5)	6 (5-6.2)	6 (5-7)	5 (4-6)	5 (3.7-6)	5 (4-6)
Prior training in risk prevention^†^											
Yes	18	58.3 (19.5)	6 (5-6)	5 (4-7)	6 (5-7)	5 (3-6)	6 (5-7)	6 (5-7)	5.5 (4.7-7)	4 (2-6)	4 (4-6)
No	60	68.5 (20.2)	4 (4-6)	6 (5-6.7)	6 (5-7)	4.5 (3-5.7)	5.5 (5-6)	6 (5-7)	5 (4-6)	5 (3.2-6)	4.5 (4-6)

*Kruskall-Wallis test; †Mann-Whitney U test; ‡Significance level: p≤ 0.05

B1: Knowledge of students of the biological risk; B2: Knowledge of persons-in-charge of the biological risk; B3: Fear of harm from the biological accident; B4: Possibility of harm from the biological agent; B5: Severity of resulting harm; B6: Extent to which the risk can be avoided; B7: Possibility of control in a risky situation; B8: Extent to which a large number of people can be harmed; B9: Immediacy of consequences; B10: Overall magnitude of the perceived biological risk.

## Discussion 

Obviously, risk reduction should be a common goal among professionals who are in contact with nursing students. Many authors agree that accidents including biological risk exposure remain relatively frequent, and knowledge among nursing students is low(11-15). This matter becomes more complicated when the concept of "risk" is not universally understood, and thus, students do not precisely understand what should be reduced(23). This matter is related to the concept of risk perception, which is a subjective expression conditioned by various factors such as knowledge, values and personal beliefs(24). Some authors agree that there is a relationship between risk perception and work attitudes among professionals (19-21). However, few studies have gone beyond describing the most common perceived risks, among which are percutaneous injuries, among professional nurses(24-25). Additionally, these studies do not relate risk perception to other variables that could influence students' attitudes towards biological risk, such as work, sociodemographics, prior theoretical or practical training, adherence to preventive measures and having experienced a prior accident. This study found that students with no previous practical training had weaker perceptions of potential harms, which could be related to students' knowledge. Additionally, there was a stronger global perception of the biological risk and catastrophic potential associated with the complex and unfamiliar clinical environment that students would encounter. However, students who had previously worked in the health sector had a weaker perception of biological risk and a stronger perception of their ability to prevent or reduce harm arising from this risk, which is important because this perception may cause a feeling of invulnerability to accidents. Finally, stronger perceptions of knowledge of the risk was found among men compared to women, which could also be related to their weaker perception of the severity of the consequences. 

A potential limitation of the study is that the study population belongs to one university, and the results may not be extrapolated to the entire community of nursing students. However, we believe that this limitation does not substantially affect the results because the sociodemographic characteristics of students are similar to those of students from other universities. Another potential limitation is that data on knowledge of biological risk were not recorded, which may also influence risk perception.

## Conclusion

These results show that undergraduate nursing students' sociodemographic, employment and training variables are related to the perception of biological risk. Students with prior practical training have a stronger perception of biological risk than untrained students. Further studies that relate the perception of biological risk to other important aspects of university education, such as students' knowledge regarding the risk, the use of preventive measures during their practical training and the biological accidents suffered, are needed. In this way, specific interventions could be designed to foster a safety culture at the university, which is an added value to university education that goes beyond academic education.

## References

[1] Ortiz S (2003). Riesgos biológicos de los estudiantes de enfermería. Enferm Clín.

[2] Tarantola A, Abiteboul D, Rachline A (2006). Infection risks following accidental exposure to blood or body fluids
in health care workers a review of pathogens transmitted in published
cases. Am J Infect Control.

[3] Arribas Llorente JL, Campins Martí M, Hernández Navarrete MJ (2005). Estudio y seguimiento del riesgo biológico en el personal sanitario. Proyecto
EPINETAC 1996-2002: Grupo de Trabajo EPITENAC.

[4] Bettancourt L, Muñoz LA, Barbosa MA, Fernández M (2011). El docente de enfermería en los campos de práctica clínica un enfoque
fenomenológico. Rev Enferm.

[5] Antolín R, Puialto MJ, Moure ML (2007). Situaciones de las prácticas clínicas que provocan estrés en los
estudiantes de enfermería. Enferm Global.

[6] Moridi G, Khaledi S, Valiee S (2014). Clinical training stress-inducing factors from the students' viewpoint
A questionnaire-based study. Nurse Educ Pract.

[7] Alzayyat A, Al-Gamal E (2014). A review of the literature regarding stress among nursing students
during their clinical education. Int Nurs Rev.

[8] Blomberg K, Bisholt B, Kullén A, Ohlsson U, Sundler A, Gustafsson M (2014). Swedish nursing student´s experience of stress during clinical
practice in relation to clinical setting characteristics and the organisation of
the clinical education. J Clin Nurs.

[9] Shaban IA, Khater WA, Akhu-Zaheya LM (2012). Undergraduate nursing students' stress sources and coping behaviours
during their initial period of clinical training a Jordanian
perspective. Nurse Educ Pract.

[10] López IM, Sánchez V (2005). Percepción del estrés en estudiantes de enfermería en las prácticas
clínicas. Enferm Clín.

[11] Kursun S, Arslan S (2014). Needlestick and Sharp Injuries among Nursing and Midwifery
Students. Int J Caring Sci.

[12] Eljedi A (2015). Prevalence and response to Occupational Hazards among Nursing Students
in Gaza Strip, Palestine The role of Personal Protective Equipment and Safety
Regulations. Public Health Res.

[13] Petrucci C, Alvaro R, Cicolini G, Cerone MP, Lancia L (2009). Percutaneous and mucocutaneous exposures in nursing students an
Italian observational study. J Nurs Scholarsh.

[14] de Souza-Borges F, Ribeiro L, Oliveira LC (2014). Occupational exposures to body fluids and behaviors regarding their
prevention and post-exposure among medical and nursing students at a Brazilian
Public University. Rev Inst Med Trop.

[15] Gir E, Caffer J, Elaine S, Silva SR, Hayashida M, Artioli A (2008). Accidents with biological material and immunization against hepatitis
B among students from the health area Rev. Latino-Am. Enfermagem.

[16] Castillo S, Vessoni RD (2007). La relación tutor-estudiante en las prácticas clínicas y su influencia
en el proceso formativo del estudiante de Enfermería. Educare 21.

[17] López C, Limón E, Castillo E, López T, Gudiol C, Isla P (2006). Precauciones estándar ¿se conocen ¿se aplican?. Rev Rol Enferm.

[18] López C, Limón E, Oto I, Carratala J, Espasa JE, Lozano V (2009). Actitudes y creencias en los estudiantes del campus de Bellvitge sobre
las medidas higiénicas y las precauciones estándar. Cultura Cuidados.

[19] Alcántara-Luque R, Rodríguez-Borrego MA, González-Galán C, Calpés-Roldán C (2013). Percepción de riesgo en alumnos de Enfermería. Enferm Global.

[20] Wojciechowski MC, Zepka L, Riegert M, Silva CF. (2012). Percepción de los estudiantes de enfermería acerca de su protección
ante patologías inmunoprevisibles. Enferm Global.

[21] Velázquez Y, Medellín J (2013). La percepción de riesgos como factor causal de accidentes
laborales. Seguridad Salud Trabajo.

[22] Cordeiro R (2002). Suggestion of an inverse relationship between perception of
occupational risks and work-related injuries. Cad Saúde Pública.

[23] Ministerio de Trabajo y Asuntos Sociales (ES). Instituo Nacional de
Seguridad e Higiene en el Trabajo (2001). NTP 578: Riesgo percibido: un procedimiento de evaluación.

[24] Fiandra U, Raciti I, Mosso R, Calipari G, Rapellino M (2008). The perception of health care risk patients, health care staff and
society. Blood Transfus.

[25] Cezar Vaz MR, Soares JFS, Figueiredo PP, Azambuja EP, Sant'Anna CF, Costa VZ (2009). Risk perception in family health work study with workers in Southern
Brazil. Rev. Latino-Am. Enfermagem.

